# Ca^2+^-regulatory proteins in cardiomyocytes from the right ventricle in children with congenital heart disease

**DOI:** 10.1186/1479-5876-10-67

**Published:** 2012-04-02

**Authors:** Yihe Wu, Wei Feng, Hao Zhang, Shoujun Li, De Wang, Xiangbin Pan, Shengshou Hu

**Affiliations:** 1 State Key Laboratory of Cardiovascular Medicine, Cardiovascular Institute & Fuwai Hospital, Chinese Academy of Medical Sciences & Peking Union Medical College, Beijing, China; 2 Center for Pediatric Cardiac Surgery, Cardiovascular Institute & Fuwai Hospital, Chinese Academy of Medical Sciences & Peking Union Medical College, Beijing, China; 3 Research Center for Cardiac Regenerative Medicine of the Ministry of Health, Cardiovascular Institute & Fuwai Hospital, Chinese Academy of Medical Sciences & Peking Union Medical College, Beijing, China; 4 Center for Pediatric Cardiac Surgery, Cardiovascular Institute & Fuwai Hospital, Chinese Academy of Medical Sciences & Peking Union Medical College, 167 Beilishi Road, Beijing, 100037, China

**Keywords:** Ca^2+^-regulatory proteins, Hypoxia, Hypertrophy, Immature cardiomyocytes, Right ventricle

## Abstract

**Background:**

Hypoxia and hypertrophy are the most frequent pathophysiological consequence of congenital heart disease (CHD) which can induce the alteration of Ca^2+^-regulatory proteins and inhibit cardiac contractility. Few studies have been performed to examine Ca^2+^-regulatory proteins in human cardiomyocytes from the hypertrophic right ventricle with or without hypoxia.

**Methods:**

Right ventricle tissues were collected from children with tetralogy of Fallot [n = 25, hypoxia and hypertrophy group (HH group)], pulmonary stenosis [n = 25, hypertrophy group (H group)], or small isolated ventricular septal defect [n = 25, control group (C group)] during open-heart surgery. Paraffin sections of tissues were stained with 3,3′-dioctadecyloxacarbocyanine perchlorate to measure cardiomyocyte size. Expression levels of Ca^2+^-regulatory proteins [sarcoplasmic reticulum Ca^2+^-ATPase (SERCA2a), ryanodine receptor (RyR2), sodiumcalcium exchanger (NCX), sarcolipin (SLN) and phospholamban (PLN)] were analysed by means of real-time PCR, western blot, or immunofluorescence. Additionally, phosphorylation level of RyR and PLN and activity of protein phosphatase (PP1) were evaluated using western blot.

**Results:**

Mild cardiomyocyte hypertrophy of the right ventricle in H and HH groups was confirmed by comparing cardiomyocyte size. A significant reduction of SERCA2a in mRNA (*P*<0.01) was observed in the HH group compared with the C group. The level of Ser^16^-phosphorylated PLN was down-regulated (*P*<0.01) and PP1 was increased (*P*<0.01) in the HH group compared to that in the C group.

**Conclusions:**

The decreased SERCA2a mRNA may be a biomarker of the pathological process in the early stage of cyanotic CHD with the hypertrophic right ventricle. A combination of hypoxia and hypertrophy can induce the adverse effect of PLN-Ser^16^ dephosphorylation. Increased PP1 could result in the decreased PLN-Ser^16^ and inhibition of PP1 is a potential therapeutic target for heart dysfunction in pediatrics.

## Background

Congenital heart disease (CHD) is a major birth defect around the world [1]. Cyanotic congenital heart diseases account for approximately 25% of all CHDs [2,3]. Tetralogy of Fallot (TOF) is the most common form of cyanotic CHD which involves a large ventricular septal defect (VSD), pulmonary stenosis, right ventricular hypertrophy and an overriding aorta [4]. Hypoxia and right ventricular hypertrophy (RVH) are the major pathophysiological change of TOF. Pulmonary stenosis (PS) and the associated right ventricular outflow tract obstruction (RVOTO) are acyanotic CHD with RVH. All the above CHDs offer natural human disease models for the study of right ventricular hypertrophic cardiomyocytes with or without hypoxia.

Ca^2+^ is a key component of cardiomyocyte excitation-contraction (E-C) coupling. Ca^2+^-regulatory proteins regulate intracellular free Ca^2+^ concentrations and maintain intracellular Ca^2+^ homeostasis so it is very important for E-C coupling and for myocyte contractility [5]. The most important Ca^2+^-regulatory proteins include sarcoplasmic reticulum (SR) Ca^2+^-ATPase (SERCA2a), ryanodine receptor (RyR2, the cardiac isoform of RyR), sodiumcalcium exchanger (NCX), sarcolipin (SLN), and phospholamban (PLN) [5]. Abnormal parts of Ca^2+^-regulatory proteins in ventricular hypertrophy (LVH) are well established from animal models [6]. Few, if any, studies have examined these proteins in human LVH or RVH, due to a lack of tissue availability. Bartelds and colleagues have reported pressure load can induce different functional and molecular adaptations in the right ventricle (RV) from left ventricle (LV) [7]. In addition, it is important that the reported Ca^2+^-regulatory proteins changes have not been consistent among all animal models of LVH [8,9]. Such discrepancies further emphasize that it is necessary to examine these proteins changes associated with RVH in the human heart, rather than generalizing from LVH of animal models. Furthermore, hypoxia is a severe pathophysiological condition that can induce RVH[10,11] and can also alter the expression of Ca^2+^-regulatory proteins in hearts from animal models, however there is no evidence of this in the human heart [10,12]. Therefore, research about the alteration of Ca^2+^-regulatory proteins in RVH with or without hypoxia will help to understand the cellular and molecular bases of RVH and hypoxia in the populations of children with CHD.

In the present study, heart tissues from the RV were collected from young patients during open-heart surgical repair, and then the expression of a series of important Ca^2+^-regulatory proteins, which including SERCA2a, RyR2, NCX, SLN, PLN and its phosphorylation sites PLN-Ser^16^ and PLN-Thr^17^, were analyzed at the mRNA and protein level respectively [13]. This study aims to investigate whether an altered Ca^2+^-handling system might be associated with RVH with or without hypoxia in children with CHD.

## Methods

### Patients and study design

All experiments were carried out in accordance with Council for International Organizations of Medical Sciences guidelines. Our local ethics committee (Fuwai Hospital Research Ethics Committee) approved this study, and informed consents were obtained from all patients.

In total, 75 children with CHD undergoing surgical heart defect repair were enrolled in this study. 25 patients with TOF were included in the Hypoxia and hypertrophy group (HH group); 25 who had PS without hypoxia were in the Hypertrophy group (H group). CHD only with small isolated VSD, had little effect on infants’ haemodynamics [4], so 25 children who had small isolated VSD without hypoxia or hypertrophy, served as the Control group (C group). No pulmonary hypertension in the C group was hinted by echo before surgery and evaluated by intraoperative measurements of pulmonary artery pressure (pulmonary arterial mean pressure<25 mmHg in all C group patients). The RV was described as hypertrophy by the surgeon in all H and HH groups, but was not described in all C group patients. Clinical and demographic characteristics of the patients enrolled in the study are reported in Table 1.

**Table 1:** Clinical and demographic characteristics of patients

	**C group**	**H group**	**HH group**	**P-value**
**Used in real-time PCR and western blot analysis**				
**Number of patients**	20	20	20	
**Age (months)**	24.6 ± 8.30	21.4 ± 13.4	22.4 ± 7.67	ns
**Body weight (kg)**	12.1 ± 2.48	11.1 ± 3.59	11.1 ± 1.89	ns
**Sex (female/male)**	10/10	11/9	12/8	ns
Peak systolic pressure gradient across the pulmonary valve (mmHg)*	6.5 ± 3.09	78.8 ± 40.51	86.2 ± 14.75	<0.001 vs C group
**O**_**2**_(% saturation)*	98.4 ± 1.15	98.3 ± 1.59	81.5 ± 5.49	<0.001 vs HH group
**Used for cell size and IF analysis**				
**Number of patients**	5	5	5	
**Age (months)**	17.8 ± 5.97	17.4 ± 5.81	19.0 ± 5.70	ns
**Body weight (kg)**	11.0 ± 2.21	9.6 ± 1.88	11.7 ± 1.17	ns
**Sex (female/male)**	2/3	3/2	2/3	ns
Peak systolic pressure gradient across the pulmonary valve (mmHg)*	8.2 ± 3.28	61.4 ± 9.94	79.2 ± 11.03	<0.001 vs C group
**O**_**2**_(% saturation)*	98.5 ± 0.87	97.8 ± 1.10	82.3 ± 5.94	<0.01 vs HH group

* Peak systolic pressure gradient across the pulmonary valve was evaluated by Echo before surgery; O_2_ saturation was evaluated before surgery.

C group, Control group; H group, Hypertrophy group; HH group, Hypoxia and hypertrophy group; IF, immunofluorescence.

### Heart tissue collection

Because excising obstructing muscle bands was a necessary procedure during TOF and PS repair, the myocardium specimens (60–200 mg net weight) were resected through the right ventricular outflow tract (RVOT) incision in H and HH groups. Endomyocardial biopsies of RV were obtained across the tricuspid valve from VSD patients in C group [5] and the net weight of myocardium specimens was much lower (only 30–50 mg). Tissues were then immediately immersed in liquid nitrogen for RNA and protein extraction [5]. For cell size and immunofluorescence (IF) analysis, the samples were fixed overnight in formalin and then embedded by paraffin [14].

### Measurement of cell size

Paraffin sections of RVOT tissues were stained with 3,3′-dioctadecyloxacarbocyanine perchlorate (DiO, Beyotime Institute of Biotechnology, Shanghai, China) that highlighted the cell membrane of cardiomyocytes. Surface areas of cardiomyocytes were measured using NIH Image J 1.32j software (http://rsb.info.nih.gov/ij/). Approximately 250 cardiomyocytes cells were chosen at random for the measurement of cell sizes [15].

### RNA isolation and cDNA synthesis

Deep-frozen biopsies were homogenized by a micro-homogenizer (Kimble, USA). Total RNA was isolated from each specimen using Trizol Reagent (Invitrogen, Carlsbad, CA, USA) and PureLink RNA Mini Kit (Invitrogen) according to the manufacturer recommended procedures [16]. Double-stranded complementary DNA (cDNA) was synthesized from 0.5 μg total RNA samples using PrimeScript® RT reagent Kit (TaKaRa Biotechnology Co., Dalian, China) according to the manufacturer’s instructions [16].

### Real-time PCR

Primers for the SERCA2a, RyR2, NCX, SLN, PLN and calsequestrin (CASQ) were all designed by Autoprime software (http://autoprime.de). The specificity of each primer was verified by the Basic Local Alignment Search Tool (BLAST) (http://www.ncbi.nlm.nih.gov/BLAST/)[4]. The sequences of the used primers are listed in Table 2. Gene-specific real-time PCR primers were synthesized by TaKaRa Biotechnology Co. Real-time PCR was performed in a 50 μl reaction, 96-well format using SYBR® Premix Ex Taq^TM^ II (TaKaRa Biotechnology) in an 7300 real-time PCR system (Applied Biosystems, Foster City, CA, USA) according to the manufacturer’s instructions [17]. A minimum of three independent experiments was done for each sample [16].

**Table 2:** Primers applied in real-time PCR

**Target gene**	**Forward primer**	**Reverse primer**	**Size (bp)**
**SERCA2a**	5'-GACCCACGAGCTGTCAACCA-3'	5'-GGATCTTGCCAATTTCGGTGTTA-3'	121
**RyR2**	5'-CCGGAAACAGTATGAAGACCAGCTA-3'	5'-CACACAACGCTGGCAATTCAC-3'	145
**NCX**	5'-TTCGTCGCACTTGGAACATCA-3'	5'-ATGGAGGCGTCTGCATACTGG-3'	83
**SLN**	5'-GGAGTTGGAGCTCAAGTTGGAGAC-3'	5'-GAACTGCAGGCAGATTTCTGAGG-3'	129
**PLN**	5'-CAACTGTTCCCATAAACTGGGTGA-3'	5'-AAGCTGGCAGCCAAATATGAGATAA-3'	152
**CASQ**	5'-GGGAGAAGACTTTCAAGATTGACCT-3'	5'-CAGAAAGCACATCCTCAATCCA-3'	150

The relative amount of target mRNA normalized to CASQ was analyzed by using the 2^-△Ct^ method [18]. CASQ is a calcium storage protein of the SR with unchanged expression on mRNA and on the protein level in both animal models of cardiac hypertrophy and hypoxia in heart failure and patients with cardiovascular disease [4,19].

### Western blot analysis

The protein expression levels of SERCA2a, RyR2, NCX, PLN, phosphorylation state of RyR (PRyR), phosphorylation state of PLN (PLN-Ser^16^ and PLN-Thr^17^), protein phosphatase (PP1) and CASQ were evaluated by western blot analysis. By using the NuPAGE electrophoresis system (Invitrogen), electrophoresis and blotting of the proteins were performed according to the instructions of the manufacturer [5]. NuPAGE® Novex 4–12% Bis-Tris Gel were used with MES or MOPS buffer. After blotting, PVDF membranes were stained with ponceau red (Beyotime Institute of Biotechnology) and then photographed (ChemiDoc XRS, Bio-Rad, Hercules, CA, USA). Membranes were blocked for 1 h with Tris–HCl 10 mmol/L, NaCl 150 mmol/L, pH 7.4 buffer (TBS) containing 5% non-fat dry milk and 0.1% Tween 20, incubated overnight with the first antibody (SERCA2a, Abcam, Cambridge, MA, USA; RyR2, Affinity BioReagents, Waltham, MA, USA; NCX, Abcam; PLN, Affinity BioReagents; PRyR-S2808, Abcam; PLN-Ser^16^, Millipore, Billerica, MA, USA; PLN-Thr^17^, SantaCruz Inc., CA, USA; PP1-alpha, Millipore; CASQ, Abcam) diluted in a specific concentration with TBS containing 5% non-fat dry milk, washed six times with TBS containing 0.1% Tween 20 (TTBS), and then incubated for 1 h with the secondary antibody labelled with peroxidase (1:10000, goat anti-rabbit IgG, Sigma-Aldrich, St. Louis, MO, USA; 1:10000, goat anti-mouse IgG, Sigma-Aldrich). Membranes were then washed with TTBS and developed with a chemiluminescent substrate (Immobilon Western Chemiluminescent HRP Substrate, Millipore). Quantification of immunoblots was done by scanning on ChemiDoc XRS (Bio-Rad) using Quantity One software [5]. CASQ was used as internal standard [4,19].

### Immunofluorescence

The protein expression level of SLN was evaluated by IF on paraffin sections. Slides were rinsed 3 times in phosphate-buffered saline (PBS, Invitrogen), blocked with 5% bovine serum albumin (Invitrogen) for 45 min followed by 3 rinses in PBS. Then sections were incubated overnight with primary antibody against SLN (1:1000, SantaCruz Inc.). The following day, slides were washed 3 times in PBS and incubated with a secondary red fluorochrome-conjugated rabbit anti-goat antibody (1:1000 dilution; Invitrogen) for 1 h at room temperature. After rinsing in PBS, The nuclei were stained with 4′,6-diamidino-2-phenylindole (DAPI, Sigma-Aldrich) for 5 min at room temperature and then pictures were taken using a fluorescence microscope (OLYMPUS BX61, Olympus Corporation, Tokyo, Japan) [20,21]. There were five patients used for SLN immunofluorescence analysis in every group. Three paraffin sections were made in every patient and three fields were evaluated in every paraffin section [5].

### Statistical analysis

Data are expressed as mean ± SD. All the data of the patients characteristics, the cell size, the gene expression, western blot and IF were analyzed by one-way analysis of variance (ANOVA). If the test of homogeneity of variances indicated that there was heterogeneity of variance, the Welch test was used for robust tests of equality of means. The least significant difference (LSD) or Dunnett T3 test was used followed by post hoc analysis with multiple comparison test to control the increase in the type I error. The statistical analysis was performed with SPSS 13.0 statistical software (SPSS, Inc., Chicago, IL). A value of *P* < 0.05 (2-tailed) was considered statistically significant.

## Results

### Patients characteristics

Age, body weight, and sex were not significantly different among the three groups (Table 1). All the patients were younger than 3 years (36 months). The peak systolic pressure gradient across the pulmonary valve in both H and HH groups was significantly higher than that in the C group, and the percutaneous O_2_ saturation in HH group was the lowest in the three groups (Table 1).

### Comparison of cardiomyocyte size

**Figure 1:**
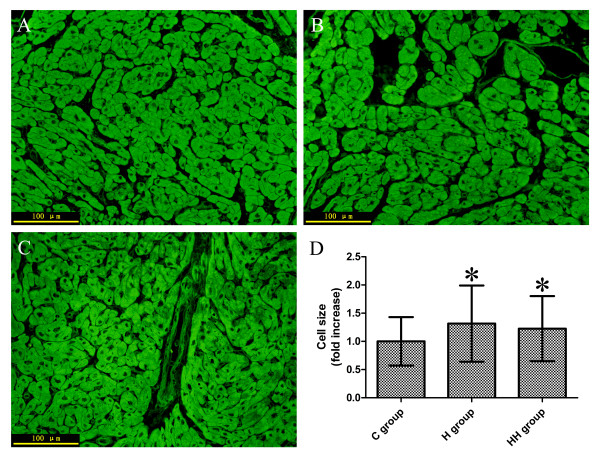
**Cardiomyocyte size quantification.** (**A, B, C**). Paraffin sections of right ventricular tissues were stained with 3,3′-dioctadecyloxacarbocyanine perchlorate that highlighted the cell membrane of cardiomyocytes (**A**. Control group, **B**. Hypertrophy group, **C**. Hypoxia and hypertrophy group. cropped from × 400 magnification); (**D**). Comparision of cardiomyocyte size (*P<0.001 vs C group). C group, Control group; H group, Hypertrophy group; HH group, Hypoxia and hypertrophy group.

A slight increase in cardiomyocyte size was found in H (+31%, *P*<0.001 *vs* C group) and HH groups (+22%, *P*<0.001 *vs* C group) when compared with C group (Figure 1).

### mRNA expression of Ca^2+^-regulatory proteins

**Figure 2:**
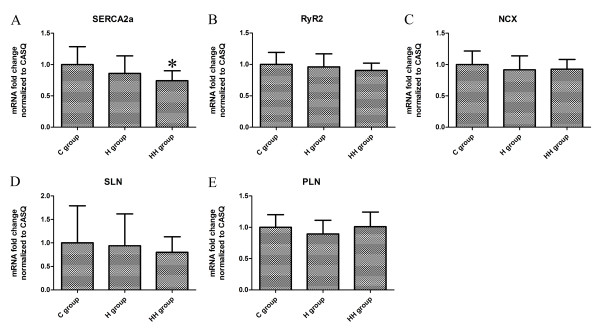
**Real-time PCR analysis of Ca**^**2+**^**-regulatory proteins.** Sarcoplasmic reticulum Ca^2+^-ATPase showed a significant decrease in mRNA levels in the HH group when compared with C group (*P<0.01 vs C group) (**A**). No significant changes were observed for ryanodine receptor (**B**), sodiumcalcium exchanger (**C**), sarcolipin (**D**) and phospholamban (**E**) expression. SERCA2a, sarcoplasmic reticulum Ca^2+^-ATPase; RyR2, ryanodine receptor; NCX, sodiumcalcium exchanger; SLN, sarcolipin; PLN, phospholamban; C group, Control group; H group, Hypertrophy group; HH group, Hypoxia and hypertrophy group.

SERCA2a, as a key regulator of intracellular Ca^2+^, in the HH group showed a decreased transcription level when compared with the C group (*P* = 0.002). However, although the similar trend of decrease was detected in the H group in comparison to the C group, there was not a statistically significant difference (Figure 2A).

The mRNA expression levels of RyR2 (Figure 2B), NCX (Figure 2C), SLN (Figure 2D), and PLN (Figure 2E) were not significantly different among the three groups.

### Protein expression of Ca^2+^-regulatory proteins

**Figure 3:**
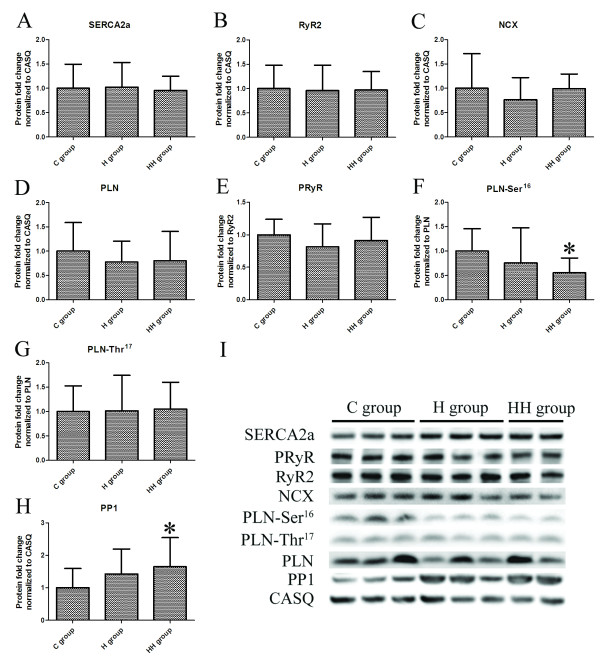
**Western blot analysis of Ca**^**2+**^**-regulatory proteins, phosphorylation level of RyR and PLN, and PP1.** The bar graphs of protein expression of SERCA2a (**A**), RyR2 (**B**), NCX (**C**), PLN (**D**), PRyR (**E**), PLN-Ser^16^ (**F**), PLN-Thr^17^ (**G**) and PP1 (**H**) (*P<0.01 vs C group). (**I**). Representative graphs for western blot. Calsequestrin (CASQ) was used as internal standard; PRyR and PLN-Ser^16^ or PLN-Thr^17^ were normalized to RyR or PLN, respectively.

**Figure 4:**
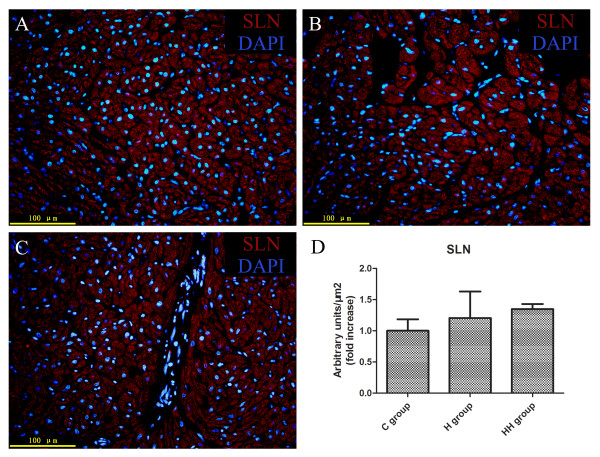
**Immunofluorescence analysis of sarcolipin.** Representative micrographs (cropped from × 400 magnification) showing there were no statistically significant change of sarcolipin in C group (**A**), H group (**B**) and HH group (**C**). Nuclei are stained blue with DAPI and sarcolipin is stained red with phalloidin conjugated with TRITC. (**D**). Semi-quantitative analysis after immunostaining of sarcolipin.

By using western blot analysis, the protein expression levels of SERCA2a, RyR2, NCX, PLN, and CASQ were evaluated. Unfortunately, the protein amount of SERCA2a was unaltered in the H and HH groups when compared with the C group (Figure 3A and I). Protein levels of other proteins including RyR2 (Figure 3B and I), NCX (Figure 3C and I) and PLN (Figure 3D and I) was also unaltered in the H and HH groups. Additionally, the relative protein expression level of SLN was obtained by IF. As shown in Figure 4, the protein amount of SLN was not significantly altered in the H and HH groups.

### Phosphorylation level of Ca^2+^-regulatory proteins

To investigate whether the phosphorylation status of RyR and PLN had been altered, PRyR, PLN-Ser^16^ and PLN-Thr^17^ were examined by western blot. There was no statistically significant difference among the three groups regarding PRyR (Figure 3E and I) and PLN-Thr^17^ (Figure 3G and I) levels. However, PLN-Ser^16^ was significantly decreased in the HH group when compared to the C group (*P* = 0.004) (Figure 3F). A similarly decreased trend was also detected in the H group although it was not statistically significantly different (Figure 3F and I).

### Activity of protein phosphatase PP1

Because phosphorylation of PLN can be regulated by PP1 in cardiomyocytes [5], its expression was examined by means of western blot. Figure 3H showed that PP1 in the HH group was statistically significantly higher than that in C group (*P* = 0.009) (Figure 3H). A similarly increased trend was also detected in the H group but there was no statistically significant difference (Figure 3H and I).

## Discussion

Previous studies have reported that immature cardiomyocytes have characteristics of Ca^2+^-regulatory proteins which are different from that of mature cardiomyocytes [22-25]. During normal cardiac development in rabbits and rats, NCX expression is maximal near the time of birth and then declines postnatally [22,25]. Conversely, SERCA2a expression levels increased during this period [23-25]. The characteristics of Ca^2+^-regulatory proteins in immature cardiomyocytes during hypertrophy with or without hypoxia has not been studied extensively, however the results of research will be beneficial for choosing an optimal time of therapy and interpreting the development process of cardiomyocytes in children with CHD.

To our knowledge, this study could be the first effort specifically dedicated to the evaluation of the gene and protein expression of Ca^2+^-regulatory proteins on human immature cardiomyocytes from RV. There were three major findings in this study. We found that SERCA2a, as a key regulator of intracellular Ca^2+^, showed a decreased transcription level in the early stage of cyanotic CHD with hypertrophic right ventricle. We also found that a combination of hypoxia and hypertrophy can induce the adverse effect of PLN-Ser^16^ dephosphorylation in human immature cardiomyocytes. The third major finding was that the decreased PLN-Ser^16^ results from an increased PP1 which can regulate phosphorylation of PLN in cardiomyocytes.

Ca^2+^-regulatory proteins regulate intracellular free Ca^2+^ concentrations and maintain intracellular Ca^2+^ homeostasis and thus have a very important role in the process of myocardial contraction and relaxation [5]. Hypoxia and hypertrophy are the most frequent pathophysiological consequence of CHD which can induce the alteration of parts of Ca^2+^-regulatory proteins and thus inhibit cardiac contractility [10,12,26]. The changes of RV are the key pathologic changes of CHD [27]. However, few studies have been performed to examine Ca^2+^-regulatory proteins in human RV during hypoxia and hypertrophy, due to a lack of tissue sources.

We investigated the expression alteration of Ca^2+^-regulatory proteins of RV during hypoxia and hypertrophy in children disease models. Because it was difficult to obtain ventricular tissue from healthy age-matched children, we chose children with small isolated VSD as the control group. The left to right intracardiac shunt was much less in small isolated VSD, so increased pulmonary pressure did not occur [28]. Additionally, there was no right to left intracardiac shunt, so there was no hypoxia in patients with small isolated VSD [28]. Therefore, there was no pressure overload-induced ventricular hypertrophy and hypoxia in RV in patients with small isolated VSD [28]. The relative normal peak systolic pressure gradient across the pulmonary valve (Table 1), cardiomyocyte size (Figure 1), and percutaneous O_2_ saturation (Table 1) in the C group was evidence that this group was suitable to serve as the control group. A slight increase in cardiomyocyte size in H (+31%, *P*<0.001 *vs* C group) and HH groups (+22%, *P*<0.001 *vs* C group) (Figure 1) suggests that pressure overload and hypoxia can induce myocardial hypertrophy. Hyperplasia maybe also exisit in this response, however, only the contribution of hypertrophy to the increase in muscle mass was measured because of the limitation of method.

SERCA2a (the cardiac isoform of SERCA) is a Ca^2+^-ATPase pump in SR that pumps Ca^2+^ back to SR during myocardial relaxation [29]. In previous studies, reports about SERCA2a expression during hypertrophy were inconsistent in animal models [30-33]. Song et al. and Wong et al. reported that a decreased mRNA and protein level in SERCA2a was in pressure overload-induced LVH [30,31], but Carvalho et al. and Diaz et al. reported no change of it was found [32,33]. Inconsistent reports were also present about the SERCA2a expression during hypoxia [10,12,34,35]. Sharma et al. and Ronkainen et al. reported that there was a downregulation of SERCA2a gene and protein expression in cardiomyocytes during hypoxia [10,12], but Yeung et al. and Larsen et al. reported that the amounts of SERCA2a were not changed [34,35]. Different animal models and different periods of ventricular hypertrophy may partly explain these inconsistencies. Our data showed that right ventricular cardiomyocytes with hypertrophy and hypoxia were associated with decreased mRNA levels for SERCA2a and the cardiomyocytes only with hypertrophy had a similar decreased trend but this was not statistically significant (Figure 2A). This suggested that in human immature cardiomyocytes, the hypertrophy alone was not enough to induce the significant alteration of SERCA2a mRNA, which can be aggravated by hypoxia. However, these alterations at the transcript level, are not accompanied by the corresponding change in protein level (Figure 3A). Our study was consistent with an earlier study performed in adult patients with LVH [26]. Further, findings similar to ours have been reported in human heart failure [36]. Our finding of downregulation of mRNA expression without concurrent decreases in protein levels suggests an uncoupling between the two processes [26]. The regulation of translation after transcription may play an important role in this process [26]. These findings suggest that because the damage effect of decreased SERCA2a mRNA can compensate in the protein level, human immature cardiomyocytes have good adaptability to hypoxia and hypertrophy, and alterations of SERCA2a in transcript levels may be markers of the pathological process, but do not result in altered protein expression.

RyR2 is a Ca^2+^ release channel of SR and NCX is a Na^+^/Ca^2+^ exchanger in cytomembrane that can remove a part of Ca^2+^ to extracellular milieu after muscle contraction [37]. Our data show no change in the mRNA or protein expression of RyR2 and NCX in the child heart during RVH with or without hypoxia (Figure 2 and 3), similar to what has previously been shown by Yeung and others [32,34]. The two small homologous intrinsic membrane proteins SLN and PLN in the sarcoplasmic reticulum, which inhibits the activity of SERCA2a, are potentially critical regulators of cardiac contractility [5]. The data from our study also show no change of SLN and PLN in mRNA or protein level in human immature cardiomyocytes during hypertrophy with or without hypoxia (Figure 2,3 and 4). Also, no alteration of PLN in cardiomyocytes during hypoxia or hypertrophy was reported by Larsen and others [32,35]. But there was few report about SLN during hypoxia or ventricular hypertrophy. Our data demonstrate that, child RVH with or without hypoxia, is not accompanied by a significant reduction in RyR2, NCX, SLN and PLN, and this suggests that it is not suitable for improving cardiac contractility by regulating these protein expressions in the early stage of CHD only with RVH and/or hypoxia.

The functional interaction between SERCA2a and PLN is regulated by the phosphorylation of PLN. PLN can be phosphorylated at distinct sites by different protein kinases: phosphorylation at the Ser^16^ residue by protein kinase A (PKA) or at the Thr^17^ site by Ca^2+^/calmodulin-dependent protein kinase II (CaMKII) [35]. Phosphorylation of Ser^16^ in PLN can occur independently of Thr^17^ in vivo and may be a prerequisite for Thr^17^ phosphorylation during β-agonist stimulation [5]. We found that Ser^16^-phosphorylated PLN was significantly reduced in human immature cardiomyocytes during hypertrophy with hypoxia, but no change in the level of Thr^17^-phosphorylated PLN was observed (Figure 3F and G), that indicated a reduction in the PKA-dependent PLN phosphorylation during hypoxia and hypertrophy [5]. Similarly, a decreased trend of PLN-Ser^16^ was also detected in child RV only with RVH, although this was not statistically significant (Figure 3F). In the unphosphorylated state, PLN inhibits SERCA2a by lowering its Ca^2+^ affinity [5]. The inhibitory function of PLN is relieved by its phosphorylation [5]. When the amount of PLN was not altered (Figure 3D), the reduction of PLN-Ser^16^ indicates a relative increase of unphosphorylated PLN that enhances inhibition of SERCA2a and thus damages cardiac contractility. These findings have important implications for the CHD treatment. Because the adverse effect was induced by a combination of hypoxia and hypertrophy, correcting hypoxia by early surgical repair may recover the phosphorylated state of PLN and contribute to the improved cardiac contractility in cyanotic congenital heart diseases with RVH.

The abnormalities in the PKA-dependent phosphorylation pathway could result from an increased PP1 [5]. Several lines of evidence demonstrate that PP1 dephosphorylates PLN-Ser^16^[38]. The PP1 catalytic subunits consist of three distinct genes, PP1α, PP1β, and PP1γ [39]. A previous study revealed that the PP1α transcript appeared to be the highest in the heart among the three isoforms, followed by PP1γ and then PP1β [39]. Therefore, in this study, we only examined the expression of PP1α by means of western blot. Our present study provides the first evidence of increased PP1 with decreased PLN-Ser^16^ in child RV during hypertrophy with hypoxia (Figure 3F and H). Boknik et al. reported PP1 activity was increased after long-term beta-adrenergic stimulation [40]. Furthermore, beta-adrenergic receptor activation can be induced by both hypoxia and hypertrophy [41-43], which can explain why the co-effect of hypoxia and hypertrophy can induce the significant increase of PP1 in human immature cardiomyocytes. Overexpression of PP1 observed in heart failure was associated with dephosphorylation of PLN, depressed cardiac function, dilated cardiomyopathy, and premature mortality [44]. Enhancement of cardiac function and suppression of heart failure progression by inhibition of PP1 were successfully done in the transgenic mice [45]. The present findings suggest regulating PLN phosphorylation by inhibiting PP1 may improve cardiac function when the right heart failure occurs in cyanotic congenital heart diseases with RVH.

### Study limitations

To study the change of all PP1 isoforms, the actual PP1 activity and its upstream regulator such as Inhibitor-1 will help us to further understand the mechanism of decreased PLN-Ser^16^ in cyanotic CHD with RVH. However, because the collected heart tissue from the sick kids was quite limited (especially in the Control group), we didn’t examine these items. We plan to investigate them in our future study.

In addition, the study is a cross-sectional study in children aged 1 month to 3 years, therefore whether the conclusions is suitable for neonatal or adult CHD requires further research.

## Conclusion

The decreased SERCA2a in transcript levels may be a biomarker of the pathological process in the early stage of cyanotic CHD with RVH. Because the protein levels of Ca^2+^-regulatory proteins were not altered, regulating their expressions in the early stages of CHD may not improve cardiac contractility. Our study suggests that a combination of hypoxia and hypertrophy can induce the adverse effect of PLN-Ser^16^ dephosphorylation, and early surgical repair might accelerate the recovery of the phosphorylated state of PLN and thereby contribute to improved cardiac contractility in cyanotic CHD with RVH. Furthermore, an increased PP1 was associated with the reduction of PLN-Ser^16^, and hence, the inhibition of PP1 might improve cardiac function and could be a potential therapeutic target for right heart failure in cyanotic CHD with RVH.

## Abbreviations

ANOVA: one-way analysis of variance; BLAST: Basic Local Alignment Search Tool; CaMKII: Ca2+/calmodulin-dependent protein kinase II; CASQ: Calsequestrin; CHD: Congenital Heart Disease; CM: Cardiomyocyte; DAPI: 4′,6-diamidino-2-phenylindole; DiO: 3,3′-dioctadecyloxacarbocyanine perchlorate; H: Hypertrophy; HH: Hypoxia and Hypertrophy; IF: Immunofluorescence; LSD: Least Significant Difference; NCX: Sodiumcalcium Exchanger; PBS: Phosphate-buffered Saline; PKA: Protein Kinase A; PLN: Phospholamban; PP1: Protein Phosphatase 1; PRyR: Phosphorylation state of RyR; PS: Pulmonary Stenosis; RVOT: right ventricular outflow tract; RVOTO: right ventricular outflow tract obstruction; RyR: Ryanodine Receptor; SERCA2a: Sarcoplasmic Reticulum Ca2+-ATPase; SLN: Sarcolipin; SR: Sarcoplasmic Reticulum; TOF: Tetralogy of Fallot; VSD: Ventricular Septal Defect.

## Competing interests

The authors declare that they have no competing interests.

## Authors’ contributions

YW and WF performed research, analyzed data and wrote the paper; HZ designed research and wrote the paper; SL, DW and XP helped to collect the samples; SH participated in the design of the study. All authors read and approved the final manuscript.

## Names of grants

This study is supported by the National Basic Research Program of China (Program 973, 2010CB529500) and Innovation Research Team in Peking Union Medical College.
